# Using online attitudinal and completion test to understand the consumerś perception of probiotic dry-fermented sausage

**DOI:** 10.1016/j.heliyon.2024.e40738

**Published:** 2024-11-28

**Authors:** Marilia Silva Malvezzi Karwowski, Eliane Cristine Francisco-Maffezzolli, Evelin da Costa Boiko, Renata Ernlund Freitas de Macedo

**Affiliations:** a Laboratory of Agri-food Research and Innovation, Graduate Program in Animal Science, School of Medicine and Life Sciences, Pontifícia Universidade Católica do Paraná, Rua Imaculada Conceição 1155, Curitiba, Paraná, 80215-901, Brazil; b Graduate Program in Administration, Business School, Pontifícia Universidade Católica do Paraná, Rua Imaculada Conceição 1155, Curitiba, Paraná, 80215-901, Brazil

**Keywords:** Meat products, Functional, Food, Projective techniques, Consumption, Health

## Abstract

This study aimed to assess consumer perceptions and the connections between consumers' health-related concerns and their perceptions of probiotic fermented sausage. The study was carried out using a 4-step online questionnaire composed of: (1) identification and recruitment; (2) application of the completion test; (3) attitudinal exploration; (4) socioeconomic inquiry. The online test was applied using images simulating the shopping experience of a couple in a supermarket. The situation demonstrated an incomplete dialog between them. The response evaluation focused on identifying the key factors influencing consumers' choices when purchasing fermented sausages, both traditional and probiotic. Sixteen categories emerged from the terms mentioned by consumers that encouraged (positive) or restricted (negative) the intention to purchase fermented sausage. Three different consumer groups were formed from the attitudinal profile results (High Health Concerned – HHC, Moderate Health Concerned – MHC, and Low Health Concerned – LHC). Among the positive categories, "healthy" and "curiosity" stood out in encouraging the purchase of probiotic fermented sausages. On the other hand, negative categories, which restrict the purchase of probiotic fermented sausages, were primarily attributed to consumers' lack of knowledge about probiotics and to the belief that the functional product has "unpleasant taste". Consumers with different health concerns exhibited distinctive perceptions of probiotic fermented sausage. To successfully introduce functional fermented sausage into the market, a multifaceted marketing approach is needed. Regardless of the level of health awareness, the education of consumers about the health benefits and the sensory attributes is crucial for the market of probiotic fermented sausage.

## Graphical abstract

**Figure:**
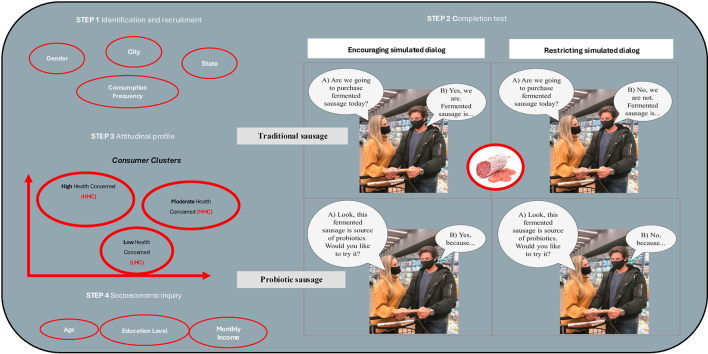

## 1 Introduction

Consumers typically perceive traditional foods as high-quality, yet simultaneously seek healthier options [1]. Fermented sausage, a traditional dry-cured product composed of minced pork, fat, spices, and often starter cultures, exemplifies this dichotomy. Similar products are produced worldwide, such as salami in Italy and Brazil and “salchichón” in Spain [2].

The established link between excessive processed meat consumption and chronic diseases like heart disease, diabetes, and cancer [3] has spurred the meat industry to innovate [[4], [5], [6]]. Consequently, reformulating meat products to reduce sodium, additives, and fat content has emerged as a promising market [[1], [2], [3], [4], [5], [6], [7]]. Moreover, the incorporation of functional attributes, particularly probiotics, into meat products has garnered increasing attention [[8], [9], [10]].

The market for functional foods is promising and, this trend will continue in the next years. A new report on “Probiotics Market-Growth, Trends, and Forecast” forecasted the global probiotics market to reach USD 76.85 billion by 2024, registering a compound annual growth rate (CAGR) of 8.15 % during the forecast period between 2020 and 2025 [11]. The processed meat market in Brazil was valued at 10.97 billion USD in 2015 (calculated in retail prices). From 2015 to 2019, it grew at a CAGR of 9.11 % to reach 15.55 billion USD, the fermented sausages share 6 % of the total [12]. Even knowing that the meat probiotic market has potential, it is essential to understand the consumer's behavior about their perceptions and attitudes toward these new functional foods [13].

There is no international consensus regarding the definition of functional foods and each country defines its own regulation [14]. In Europe, the European Commission's Concerted Action on Functional Food Science (FuFoSE) defined that: ''a food product can only be considered functional if, together with its basic nutritional impact, it has beneficial effects on one or more functions of the human organism, either improving the general and physical conditions or/and decreasing the risk of the evolution of diseases. The amount of intake and form of the functional food should be as it is normally expected for dietary purposes. Therefore, it could not be in the form of a pill or capsule but only as a normal food form. Brazilian law does not allow the use of the term “functional” on the label of functional food. Nonetheless, the legislation states that functional food labels must contain a word or a sentence indicating the physiological effect or beneficial health effect provided by the functional ingredient in the food. The label word or sentence must be previously approved by the Brazilian National Sanitary Surveillance Agency (ANVISA) [15].

In the last decades, sensory science has been widely studied to help consumers accept or reject food products. This increase in sensory evaluation grew rapidly together with the growth of the food industry [16]. Two primary methods can be used for assessing consumer choices. The first, revealed preference, observes actual consumer behavior in real-world market conditions. Conversely, stated preference presents consumers with hypothetical scenarios to evaluate potential products not yet available in the market [17]. The latter approach is particularly valuable for predicting product acceptance and informing launch decisions [18]. Emotional and unconscious reactions can be assessed using different approaches such as focus groups, joint analyses of factors, and projective techniques [[19], [20], [21]]. Projective techniques, such as word association, completion tests, and shopping lists, have become usual and widely used to know consumers' perceptions, attitudes, motivations, and needs [22]. Projective techniques are useful for understanding the consumer's perceptions of new ideas. It is possible to predict how they think and what they feel. Moreover, consumers are stimulated to be more creative, and liberal. It is possible to access deep attitudes and emotions, which reveal non-conscious behavior and help consumers overcome preconceptions [23]. The completion test consists of presenting a sentence, story, argument, or conversation (question) that can be shown in images in a comic strip and must be completed (answer) by the consumers [24]. Additionally, the completion test is a valuable tool for eliciting consumer insights through online questionnaires [25]. Projective techniques have been used to obtain sensory insights on meat products [[25], [26], [27]], however, the use of those techniques for functional meat products still needs to be assessed.

Many factors influencing consumer expectations of meat quality have yet to be fully identified, ranked, or their interrelationships explored. Consumer perception of fermented sausage is influenced by various factors such as flavor, health implications, innovation, local origin, and information labels [28]. Flavor is a complex stimulus involving taste, odor, texture, and temperature. The meat, salt, lactic acid, fat, spices, and chemical preservatives are major contributors to flavor [29]. However, the presence of nitrite has displayed harmful health effects and negatively influenced consumer perception [20,22,28,30,31].

Technological innovations have been applied in the meat industry, however with limited focus on consumer perception of these innovative products, particularly concerning their association with traditional production methods and human health [31]. This has led to some consumer skepticism regarding the innovation of traditional products when exclusively associated with health attributes. Moreover, the intention to purchase functional foods is influenced by the consumer's familiarity with the ingredient, i.e., acceptance will be greater in cases in which consumers know the benefits of the food for their health [20].

In this context, the meat industry faces significant challenges and must adapt to the growing demand for healthier and clean-label products [32]. The global market for clean-label ingredients is projected to reach $51.14 billion by 2024 [33]. In response to this trend, substantial global investments have been directed toward the exploration of novel ingredients and the study of consumer perceptions regarding clean labels and healthier products [32,34,35].

The need to preserve tradition amidst the increasing demand for healthier meat products has prompted us to explore consumers’ perceptions of traditional and functional dry-fermented sausage. Therefore, this study aimed to understand the consumers' perceptions of traditional and probiotic fermented sausage and to assess the relationships between those perceptions and the consumer's concerns about their health.

## 2 Material and methods

This exploratory study employed a survey methodology, utilizing a structured interview script administered via an online questionnaire hosted on Google Forms. Participants were Brazilian consumers aged 18 years or older who volunteered to participate anonymously without receiving compensation. Recruitment was conducted through major social media platforms. Prior to survey commencement, participants provided informed consent, accessible on the Google Forms platform. The questionnaire's structure was informed by previous research [25,36,37]. Online tools are easy to apply due to the popularization of the internet, collecting data in a wide way, diversified, fast, and reduced cost compared to traditional sensory research techniques. A pilot test was conducted on a small group of 10 consumers to assess the questionnaire's clarity and identify any errors requiring correction before finalization. Data collection then proceeded using a convenience sample of consumers who completed a four-step online questionnaire hosted on Google Docs (www.docs.google.com). The questionnaire comprised the following sections: (1) identification and recruitment; (2) completion tests; (3) attitudinal exploration; and (4) socioeconomic inquiry [36,38].

Approval for human use was granted by the Ethics Research Committee (CEP) of the Pontifical Catholic University of Paraná (PUCPR) according to (CAAE: 51290621.0.0000.0020, number 4.977.554).

### 2.1 Identification and recruitment (step 1)

This section was composed of four questions. The first three questions addressed gender [optional], state [mandatory], and city [mandatory]). The fourth question, a multiple-choice question, was about the frequency of fermented sausage consumption (mandatory): [ (always, frequently (2 or 3 times a month), moderately (once a month), sometimes (less than once a month), rarely (special occasions)].

### 2.2 Application of the completion test (step 2)

**Fig. 1:**
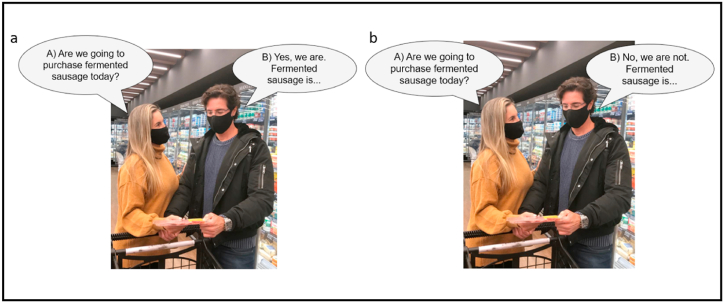
Scenes of simulated dialog used for completion test to assess features that encourage (a) and restrict (b) the purchase of traditional fermented sausage.

**Fig. 2:**
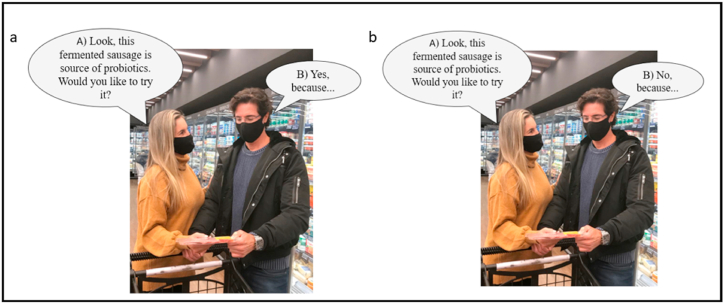
Scenes of simulated dialog used for completion test to assess features that encourage (a) and restrict (b) the purchase of probiotic fermented sausage.

After completing the previous task (step 1), the participants were directed to a new session in the questionnaire (step 2). The completion test was composed of four dialogues with different stimuli. Dialogues were shown in pictures simulating a daily situation in which a married couple is at the refrigerated meat section is a market reading the product label and having a conversation about it. The dialog was represented by speech bubbles identified with the letter "A" for the question and the letter "B" for the answer. The respondents should complete the answer with the first terms or sentences that came to their minds The first and second dialogues assessed features that encourage and restrict the purchase of traditional fermented sausage, respectively (Fig. 1a and b). The third and fourth dialogues assessed the features that encourage and restrict the purchase of functional fermented sausage, respectively (Fig. 2a and b). The sentences were presented following [25,39]. Each image represented a session within the questionnaire, and each session was presented to the consumer individually. The consumer completed the test associated with the first image before gaining access to the test related to the second image.

The associations (statements and/or words) provided by the respondents were analyzed with the search for recurring terms for each situation (features that encourage and restrict the purchase of traditional and functional fermented sausage). After, three independent researchers grouped similar terms in the same category with a personal interpretation of the meanings and synonyms of the words. Subsequently, the three researchers independently discussed the categorization, and the names of the final categories were obtained by consensus [25].

### 2.3 Attitudinal profile (step 3)

After finishing the previous task (step 2), participants were guided to a new session in the questionnaire (step 3). The participants were asked to answer a questionnaire on how much they agreed with a list of sentences about their attitude towards health. The sentences were obtained from the Health Consciousness Scale (HCS) questionnaire [40]. This questionnaire is used to assess how much the population is ready and willing to act and keep one habit of living healthy and consists of 11 sentences, of which, seven are related to worries about health and four to no concerns about healthy habits (Table 2). The questionnaire was translated and validated into Portuguese by Ref. [41], making it suitable for use with Brazilian consumers. A nine-point scale was applied, with scores ranging from (1) “strongly disagree” to (9) “strongly agree”.

### 2.4 Socioeconomic inquiry (step 4)

In the last step, three questions were used to classify the respondents according to their socioeconomic profile. The questions addressed the age, level of education, and monthly income of the respondents. The social class classification was carried out according to the criteria of the Brazilian Institute of Geography and Statistics [42].

### 2.5 Statistical analysis

A cluster analysis was used to identify groups of consumers with different health behaviors. Agglomerative hierarchical cluster analysis was performed based on the results of the attitudinal questionnaire using Ward's method and the Euclidean distance. Significant differences among clusters were identified using Analysis of Variance (ANOVA) and Tukey's post-hoc test (p < 0.05). The χ2 test was used to assess differences among consumer clusters for positive- and negative-terms categories. Statistical analysis was performed using SPSS 23.0 software (Chicago, IL).

## 3 Results and discussion

### 3.1 Respondents

A total of 233 participants answered the questionnaire. However, 32 respondents were excluded due to incomplete or inconsistent responses, resulting in a final sample of 201 participants for analysis. The socioeconomic profile of the respondents is shown in Table 1.

**Table 1:** Socioeconomic profile of consumers.

Category	Total (n = 201)	%
Gender		
Female	157	78
Male	44	22
Age group (years)		
18 - 25	38	19
26 - 35	46	23
46 - 56	42	21
36 - 45	59	29
57 - 70	15	7
> 70	1	0
School level		
Secondary incomplete	2	1
Secondary	23	11
Undergraduate incomplete	29	14
Undergraduate	55	27
Graduate	92	46
Monthly income (US$)		
≤440.00	18	9
≥440.01 ≤ 896.00	39	19
≥896.01 ≤ 2200.00	72	36
≥2200.01 ≤ 4400.00	48	24
>4400.01	24	12

∗ Brazilian 2023 minimum wage per month = BRL 1320.00 ≈ USD 270.00.

**Table 2:** Sentences in the scale of self-awareness regarding health and the average score for the different consumer clusters.

Statement	Consumer clusters			
	High health concerned (HHC) (n = 68)	Moderate health concerned (MHC) (n = 84)	Low health concerned (LHC)(n = 49)	
	Average score			p-value
1. I have the impression that I sacrifice a lot for my health.	5.19 ± 0.30^a^	5.23 ± 0.23^a^	4.27 ± 0.27^b^	0.035
2. I consider myself very health conscious.	8.12 ± 0.11^a^	7.10 ± 0.14^b^	5.18 ± 0.24^c^	0.000
3. I am prepared to sacrifice a lot, to eat as healthy as possible.	7.9 ± 0.13^a^	6.74 ± 0.16^b^	4.57 ± 0.25^c^	0.000
4. I think that I take health into account a lot in my life.	8.15 ± 0.11^a^	6.87 ± 0.17^b^	4.98 ± 0.20^c^	0.000
5. I think it is important to know well how to eat healthily.	8.75 ± 0.06^a^	8.38 ± 0.11^a^	6.98 ± 0.24^b^	0.000
6. My health is so valuable to me that I am prepared to sacrifice many things for it.	7.46 ± 0.16^a^	6.99 ± 0.17^a^	4.69 ± 0.23^b^	0.000
7. I have the impression that other people pay more attention to their health than I do.	4.03 ± 0.30^c^	6.48 ± 0.22^b^	7.55 ± 0.24^a^	0.000
8. I do not continually ask myself whether something is good for me.	2.87 ± 0.24^b^	5.2 ± 0.28^a^	5.51 ± 0.31^a^	0.000
9. I don't often think about whether everything I do is healthy.	2.81 ± 0.21^b^	5.35 ± 0.26^a^	5.78 ± 0.32^a^	0.000
10. I don't want to ask myself all the time, whether the things I eat are good for me	3.13 ± 0.23^c^	5.60 ± 0.26^b^	6.78 ± 0.28^a^	0.000
11. I often dwell on my health.	7.84 ± 0.18^a^	6.90 ± 0.14^b^	4.69 ± 0.22^c^	0.000

Different lowercase letters in the same row indicate significant differences among clusters (p < 0.05).

### 3.2 Attitudinal questionnaire

Three distinct clusters emerged as described below based on the responses collected in the attitudinal questionnaire (Table 2).

High Health Concerned Cluster (HHC): this cluster exhibited a strong awareness of their health, providing significantly higher scores (p < 0.05) for statements 1, 2, 3, 4, 5, 6, and 11, while scoring significantly lower (p < 0.05) for statements 7, 8, 9, and 10. This cluster can be characterized as highly health-conscious consumers.

Moderate Health Concerned Cluster (MHC): the MHC displayed a moderate level of agreement with statements 1, 2, 3, 4, 5, 6, and 11, and gave the highest scores (p < 0.05) to statements 8, 9, and 10. This group maintains a moderate interest in foods with health-related attributes.

Low Health Concerned Cluster (LHC): the LHC cluster consistently scored the lowest averages (p < 0.05) for statements 1, 2, 3, 4, 5, 6, and 11. This indicates that respondents in this cluster are less concerned with and less motivated to prioritize their health.

Table 3 shows the socioeconomic profile of consumers categorized in the different clusters. MHC cluster is composed of 84 consumers (41.8 % of 201 consumers), HHC cluster of 68 consumers (33.8 %), and LHC cluster of 49 consumers (24.4 %). All clusters were composed mainly of women (HHC cluster = 79 %, MHC cluster = 69 %, and LHC cluster = 90 %).

**Table 3:** Socioeconomic profile of consumers categorized in clusters according to the attitudinal responses.

Category	High health concerned (HHC Cluster) (%)	Moderate health concerned (MHC Cluster) (%)	Low health concerned (LHC Cluster) (%)	Total (n = 201) %	p-value
Gender					0.028^a^
Male	31	21	10	22	
Female	69	79	90	78	
Age group (years)					0.398
18 - 25	21	14	24	19	
26 - 35	24	20	27	23	
46 - 56	16	23	24	21	
36 - 45	31	35	18	29	
57 - 70	0	8	4	7	
> 70	0	0	2	0	
School level					0.179
Secondary incomplete	0	3	0	1	
Secondary	17	6	9	11	
Undergraduate incomplete	12	16	9	14	
Undergraduate	32	22	30	27	
Graduate	39	53	52	46	
Monthly income (US$)					0.345
≤440.00	9	6	14	9	
≥440.01 ≤ 896.00	16	20	22	19	
≥896.01 ≤ 2200.00	28	42	37	36	
≥2200.01 ≤ 4400.00	31	21	18	24	
>4400.01	16	11	8	12	
Frequency of consumption					0.080
Rarely	49	29	22	34	
Sometimes	13	33	33	26	
Moderately	15	21	12	17	
Often	18	8	22	15	
Ever	6	8	10	8	

a Significant difference among clusters (p < 0.05).

Generally, women tend to exhibit greater concern for their own health compared to men. Some studies have reported that women often avoid unhealthy diets and adopt healthier lifestyle behaviors, including regular exercise and sufficient sleep [26,27]. However, interestingly, in the present study, the LHC cluster, which comprised a larger proportion of women, emerged as the least health-conscious consumer group. The literature on gender-based attitudes towards healthy food products and their attributes has yielded divergent results. Some studies suggest that gender may have a non-significant role in this context [43]. However, other research indicates that women consider nutritional labels more important than men when selecting meat products and are more willing to pay a premium for healthier options [44]. Conversely, other studies have shown that men may be more satisfied with the healthy attributes of certain products, such as dry-cured ham [31].

Regarding the frequency of consumption of fermented sausage, 34 % of consumers reported the consumption as “rarely” (only on special occasions), 26 % as “sometimes” (less than once a month), 17 % as “moderately” (once a month), 15 % as “often” (two to three times a month) and 8 % as “always” (once a week or more).

### 3.3 Completion test

The mentioned terms were grouped into 16 categories, nine positive, and seven negative categories according to the questions that restricted (negative) or encouraged (positive) the intention to purchase traditional or functional fermented sausage.

The main terms mentioned by the consumers are described in Table 4.

**Table 4:** Description of terms according to positive and negative categories.

Positive categories	Mentioned terms
Good flavour	Yummy; Tasty, Delicious
Consumption preferences	Appetizer; Snack; Great for sandwiches; Good for snacks
Affordable price	It is cheap; It is inexpensive
Special occasion	It is Friday; It is a special day; We will receive a visit; To make a dinner/grill barbecue
Event menu	Appetizer/grazing; Table/starter for a special dinner with friends/family
Healthy	It's functional; It contains probiotics; It's good for health; It improves digestion; It's good for the intestinal flora; It's good for the body
Curiosity	I like to try new things; It is different; It seems like a good choice; It seems like a good idea; It's a new release; Looks interesting; I've never seen fermented sausage with probiotics; Probiotic is a trend
Taste	It is tasty and now it contains probiotics; It brings health benefits and is tasty
Desire	I want it
Negative categories	Mentioned terms

Rejection	I don't want it
Unhealthy	It is salty; It contains additives, It is carcinogenic
Unpleasant flavor	It tastes bad; It's not tasty
Disinterest	I don't think it's necessary to buy it; We don't need it; We already have it at home
Prefer traditional	I don't know the origin/brand; I prefer traditional/regular
Expensive	It is expensive
Probiotic unawareness	I don't know what probiotics are; What are probiotics?; How does it work?

No significant differences were observed among clusters concerning the reasons indicated by consumers as encouraging the purchase of dry-fermented sausage (χ2 = 6.57, p = 0.765). A similar pattern was observed with functional dry-fermented sausage, where no significant differences were identified among consumers regarding the characteristics that encourage the purchase of functional dry-fermented sausage (χ2 = 16.06, p = 0.488) (Table 5).

**Table 5:** Number of terms mentioned in the completion test for the situations that encourage (positive) and restrict (negative) the purchase of traditional and functional dry-fermented sausage by the different consumer clusters.

Positive Categories	Traditional dry-fermented sausage					Functional dry-fermented sausage				
	HHC Cluster	MHC Cluster	LHC Cluster	*X*^*2*^	p-value	HHC Cluster	MHC Cluster	LHC Cluster	*X*^*2*^	p-value
				6.57	0.765				16.06	0.448

Good flavour	24	27	15			7	7	2		
Consumption preferences	14	21	9			0	1	0		
Affordable price	2	6	2			1	1	0		
Special occasion	11	10	9			–	–	–		
Event menu	2	6	3			–	–	–		
Desire	11	3	10			0	1	1		
Healthy	–	–	–			38	24	21		
Curiosity	–	–	–			22	34	23		
Taste	–	–	–			0	1	1		
Negative Categories										

				29.61	0.076				27.07	0.078
Rejection	2	7	4			1	4	0		
Unhealthy	40	29	19			11	11	7		
Unpleasant flavor	2	8	3			13	26	5		
Disinterest	9	6	10			7	14	6		
Prefer traditional	–	–	–			3	4	4		
Expensive	14	23	9			11	6	6		
Probiotic unawareness	–	–	–			19	11	18		

HHC - High Health Concerned; MHC - Moderated Health Concerned; LHC - Low Health Concerned.

The similarity in consumer behavior across clusters regarding the encouraging factors for purchasing both conventional and probiotic fermented sausages is not surprising. This phenomenon can be attributed to the traditional and cultural significance of fermented sausage consumption in many countries, and it aligns with the Theory of Learned Behavior [45]. Fermented sausages possess a distinctive flavor that originates from the ingredients and chemical transformations that occur during the ripening process. This flavor is recognizable to consumers due to their familiarity with this type of food, and their consumption habit has become firmly established [28]. The habit of consumption is passed down from one generation to the next, and this type of product is often associated with tasty food, enjoyed in various forms and on special occasions. The process of consumer learning involves individuals acquiring knowledge about buying and consuming products, which they subsequently utilize to shape their future actions and decisions [46].

**Fig. 3:**
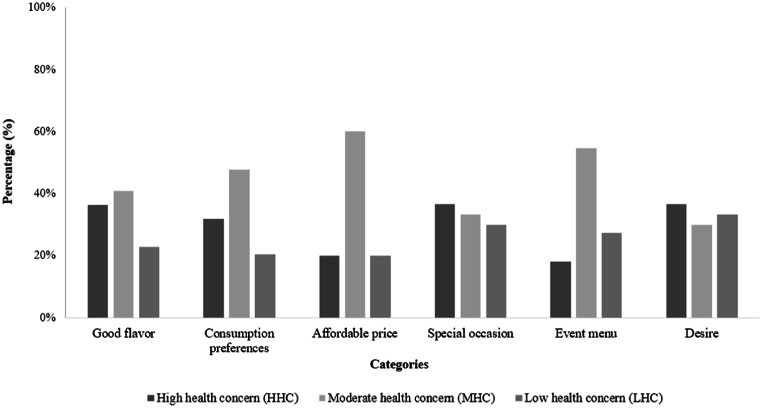
Percentage of terms mentioned in the completion test for the categories that encourage the purchase of traditional fermented sausage in the different consumer clusters.

When considering the entire consumer group (n = 201, 100 %), the leading categories that encourage the purchase of traditional fermented sausages were "good flavor" (averaging 35 %) and "consumption preferences" (averaging 23 %). Within the HHC cluster, "good flavor" was mentioned by 36 % of respondents, while the MHC cluster mentioned it in 41 % of cases, and the LHC cluster in 23 % (Fig. 3). This highlights that, for consumers of traditional fermented sausages, flavor stands out as the most crucial attribute of this product.

The consumers associated the term “good flavor” with responses that consider traditional fermented sausage “tasty”, “delicious”, and “yummy”. Sensory attributes are also considered experience attributes because consumers have an experience of evaluating the product when tasting it [47]. Flavor plays an important role in driving consumers' food choices, followed by freshness and nutritional value [48]. Therefore, regardless of the health concerns, all consumers expect a good sensory experience (good flavor and taste) when consuming fermented sausage. Similar behavior was observed by Spanish consumers who associated "high quality" with the distinct and unique sensory properties of Iberian meat products. This perception may be linked to the most prominent emotions which include feelings of being "intense," "pleasant," and "authentic" [49].

The second most relevant positive category of terms was “consumption preferences”. Within this category, terms such as "great for sandwiches" and "good for snacks" were mentioned. Additionally, the category "special occasions" emerged as another relevant category that is related to "consumption preferences," encompassing terms like "friends' gatherings", "special dinners", and "leisure moments". Both categories are closely associated with the concept of convenience, which aligns with an important trend in the food industry. They also reflect the growing demand for food options that require less preparation time [50].

Conversely, affordable price appears to be less important for consumers within the HHC and LHC clusters. In contrast, price stands out as an important factor for consumers in the MHC cluster (Fig. 3). Studies have shown that consumers are willing to pay a higher price for healthier fermented sausages [51,52]. A study on low-nitrate salami consumption found that a significant proportion of women not only preferred and consumed healthier meat products compared to men but were also willing to pay a premium price (an additional 20 %) for these products [1]. A study of Irish consumers of processed meat revealed that their purchase intentions were mainly driven by the product's price and the type of meat used. Secondary factors included the presence of ingredients perceived as healthy, as well as the salt and fat content [30].

When considering the entire consumer group (n = 201, 100 %), the leading categories that encourage the purchase of functional dry-fermented sausage were "healthy" (averaging 45 %), and "curiosity" (averaging 42 %).

The category “healthy” was associated with the terms “it is functional”, “it is good for the body”, “it improves digestion”, and “it is good for the intestinal flora”. The category “curiosity” was mentioned by consumers with the sentences: “it is a new release”, “looks interesting”, “I've never seen fermented sausage with probiotics”, and “probiotics are a trend”.

**Fig. 4:**
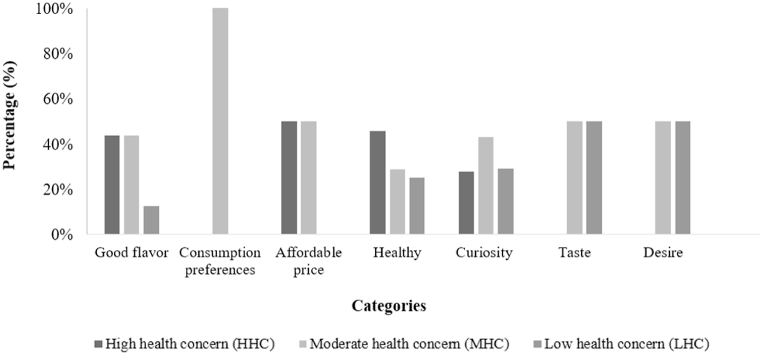
Percentage of terms mentioned in the completion test for the categories that encourage the purchase of functional fermented sausage in the different consumer clusters.

The category "healthy" was more frequently mentioned by consumers in the HHC cluster, who prioritize their health and are inclined toward healthy food choices. In contrast, consumers in the MHC cluster expressed a greater frequency of the category "curiosity" (Fig. 4). This indicates that consumers with lower health awareness tend to exhibit more curiosity in trying a new product. For these consumers, the appeal of a product with unique characteristics, distinct from conventional options, appears to hold greater importance than the potential health benefits associated with its consumption.

Currently, in the health and wellness context, a diverse range of new food products is readily accessible and designed to meet different consumer needs. This includes products enhanced with probiotics, often referred to as "functional foods," which show increasing popularity. An essential factor in the consumption of these functional foods is the pursuit of good health [29]. This connection is observed in the present study, wherein consumers belonging to the HHC cluster cited the category "healthy" more frequently. Therefore, consumers who are concerned with maintaining good health and overall well-being are more prone to show an attitude toward the willingness to consume functional foods. An evaluation of Brazilian consumer attitudes toward meat products as functional resources (dietary fiber and omega-3) revealed a positive perception, with terms such as "curiosity to taste," "functionality," and "benefits for the body" frequently mentioned [20]. In another study with South American consumers, the most prevalent categories associated with burgers added with antioxidants were “healthy” and “interest/curiosity to taste” [21] Also, the reduced sodium and/or the high omega-3 content were pointed out as the key factors influencing consumers' purchasing behavior of bologna sausage [19].

Consumer preferences for traditional and innovative pork products derived from three underutilized pig breeds—Porc Negre (Spain), Cinta Senese (Italy), and Krškopolje (Slovenia)—were assessed. Overall, traditional pork products were perceived positively across all countries, as indicated by high agreement with positive statements and low mean scores for negative attributes. However, notable disparities emerged, particularly regarding perceived health benefits. While these products were generally considered healthy in Slovenia and Spain, they received lower ratings in Italy [53].

Moreover, the trust in the label information and the knowledge of the health benefits also contribute to consumers' positive perception of a new product [31]. The importance of the label in the commercialization of meat products is a relevant topic under discussion [[54], [55], [56]]. In this study, participants expressed the need for more detailed information about new ingredients on labels, which would help reassure consumers. These results are consistent with those of [57], who examined the sensory properties of dry-cured sausages enhanced with fish oil and emphasized the impact of label information. In their study, providing clear nutritional data and statements on nutritional values and health benefits on the label led to higher test scores and increased purchase intentions, as this information is easily understood by consumers without requiring in-depth knowledge of nutrition [58].

The impacts of health motivation are particularly relevant for products with lower levels of pleasure, meaning that a higher level of healthiness motivation may compensate for a product with an inferior flavor compared with the conventional one [59]. This behavior is observed in consumers of LHC cluster who presented a lower perception of the relationship between “good flavor” and functional fermented sausage. According to Ref. [60], the curiosity to consume a product with the addition of functional ingredients is mainly related to the pleasure of exploring positive emotions, rather than to the expected health benefits.

Regarding the reasons indicated by the consumers as restricting the purchase of dry-fermented sausage, whether traditional or probiotic, marginal differences (P < 0.10) were found among the clusters (χ2 = 29.61, p = 0.076 for traditional and χ2 = 27.07, p = 0.078 for probiotic sausage) (Table 5).

The main limitations pointed out by consumers (n = 201, 100 %) for the purchase of traditional sausage, in order of importance, were represented by the categories: “unhealthy” (averaging 48 %), “expensive” (averaging 25 %), and “disinterest” (averaging 14 %).

**Fig. 5:**
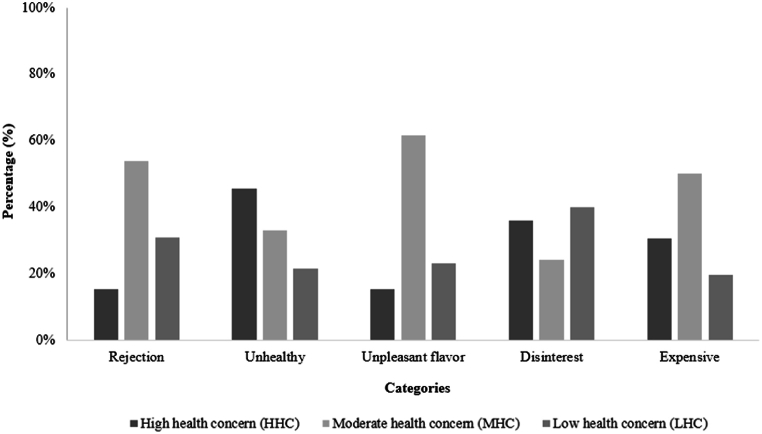
Percentage of terms mentioned in the completion test for the categories that restrict the purchase of traditional fermented sausage in the different consumer clusters.

“Unhealthy” was the most mentioned category by consumers of HHC cluster (Fig. 5). In this category, the terms “rich in sodium and fat”, “artificial”, “carcinogenic”, and “caloric” were mentioned and reflected the consumer's perception of the presence of many additives in fermented sausage, which could be assumed as the main characteristics that contribute to the negative perception of this product. Previous studies have shown that consumers believe that the levels of sodium, fat, and chemical additives present in meat product formulations are the main causes of health risks associated with the consumption of these products [61,62].

“Expensive” was the second most relevant negative category of terms mentioned by consumers that restrict the purchase of traditional fermented sausage. Consumers of the MHC cluster mentioned it at a higher frequency (Fig. 5). This finding aligns with the results obtained for the positive category "Affordable price," which showed a higher frequency of terms mentioned by the MHC cluster. It highlights the impact of price on consumers' purchase intentions and choices [48].

Regarding all consumers (n = 201) of the functional dry-fermented sausage, the main category that restricted the intended purchase was “probiotic unawares” (averaging 48 % of mentioned terms), which was associated with the sentences: “I don't know probiotics”, “what are probiotics?”, and “how does it work?”.

Other negative categories in descending order were “unpleasant flavor” (averaging 44 %), “unhealthy” (averaging 29 %), “disinterest” (averaging 27 %), and “expensive” (averaging 23 %).

**Fig. 6:**
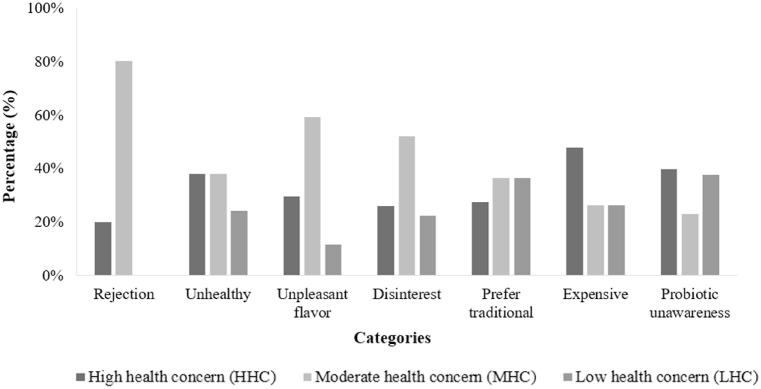
Percentage of terms mentioned in the completion test for the categories that restrict the purchase of functional fermented sausage in the different consumer clusters.

Interestingly, consumers of HHC cluster, with the highest awareness of health, showed a similar lack of knowledge about probiotic meaning as consumers of LHC cluster (Fig. 6). A study among Italian consumers revealed a limited understanding of the functional food concept [63]. Conversely, Brazilian consumers expressed a belief in the therapeutic potential of functional foods, either individually or in combination. However, skepticism regarding their efficacy also prevailed among a segment of the Brazilian population [64].

The term functional food, in some cases, is related to ‘‘light’’ and “diet” products (16 %), or even to people with health problems (9.5 %). Although, most consumers believe that functional foods improve health (34.3 %) or reduce the incidence of diseases (28.5 %).

“Unpleasant flavor” was also mentioned as a category that restricts the purchase of probiotic fermented sausage. Consumers generally associate traditional fermented sausage with a delicious flavor. However, for a new product, such as fermented sausage added with probiotics, the taste is unfamiliar and therefore, they could expect a different or a not-so-pleasant flavor.

For the MHC cluster, “disinterest” and “rejection” were also mentioned as factors that restrict the purchase of probiotic fermented sausage. It might be associated with the higher perception of “unpleasant flavor” of probiotic sausage by this consumer cluster. Nevertheless, the category “rejection” was the least mentioned by consumers of all clusters. This suggests that functional fermented sausage has a favorable opportunity for a successful launch in the market.

The results showed that for consumers of the HHC cluster, who initially considered fermented sausage as unhealthy food, the healthiness and the potential beneficial effects associated with the product are the driving factors for its consumption. On the other hand, for MHC consumer cluster, a good sensory experience is paramount for the consumption of the probiotic product. Nevertheless, consumers of all clusters were curious about the new product even if they were not fully informed about the actual impact of probiotics on their health. Therefore, specific marketing strategies are needed to conquer potential consumers with different perceptions [65,66]. Point-of-sale activities and materials (food demonstrations; taste tests; folders) are common strategies used in supermarket campaigns [67]. Manufacturers use food demonstrations and sensory tests to introduce customers to new products and increase their sales. For instance, providing samples of delicious and healthy fermented sausage can reduce the tendency of consumers to make choices solely based on the belief that unhealthy options are tastier [68]. Moreover, consumers must be informed about the health benefits of probiotic fermented sausage and the positive effects of this food must be prominently featured on the product label in a concise and easily understandable manner.

## 4 Conclusion

Consumers with different health concern profiles showed distinctive perceptions of probiotic fermented sausage. However, for all consumers, the driving factors that encouraged the intention to purchase probiotic fermented sausage were the product's association with health benefits and curiosity about the new product. On the other hand, the belief that the functional product is not as tasty as the traditional one and the unfamiliarity with the probiotic concept restricted the intention to purchase it.

Combined marketing strategies must be considered for functional fermented sausage to be accepted in the marketplace. For instance, for consumers who associate its consumption with unpleasant flavor, tasting campaigns become essential. Simultaneously, for consumers who lack awareness of probiotics' health advantages, clear labeling emphasizing these benefits becomes imperative. Healthier products are appreciated across various consumer segments. By incorporating different forms of label information, such as nutritional claims, these products can achieve effective differentiation.

It is crucial to acknowledge that the convenience sample employed in this study may not accurately reflect the broader Brazilian population. While widely used in research and often deemed reliable, this sampling method limits population representativeness. Future research would benefit from expanding the study to encompass a more diverse geographic scope, including other regions within Brazil and South America.

To further deepen the understanding of consumer preferences, future studies should explore a wider range of health-related attributes associated with fermented sausages. Specifically, investigating consumer perceptions of reduced saturated fat and sodium content, the substitution of synthetic additives with natural alternatives (aligned with the "clean label" trend), and the incorporation of biologically active substances would provide valuable insights into consumer preference hierarchies. Additionally, examining purchasing behaviors linked to other lifestyle concerns related to processed meat could offer further opportunities for research.

## CRediT authorship contribution statement

**Marilia Silva Malvezzi Karwowski:** Writing – review & editing, Writing – original draft, Methodology, Investigation, Formal analysis, Data curation, Conceptualization. **Eliane Cristine Francisco-Maffezzolli:** Writing – review & editing, Writing – original draft, Formal analysis, Data curation. **Evelin da Costa Boiko:** Methodology, Investigation, Formal analysis, Data curation. **Renata Ernlund Freitas de Macedo:** Writing – review & editing, Writing – original draft, Project administration, Methodology, Data curation, Conceptualization.

## Consent to publish

The authors affirm that human research participants provided informed consent for the publication of the images in Fig. 1, Fig. 2.

## Ethical statement

Ethical approval for the involvement of human subjects in this study was granted by Pontifícia Universidade Católica do Paraná/PUCPR, Research Ethics Committee, CAAE 51290621.0.0000.0020, number 4.977.554. The study was carried out using an online questionnaire. All participants acknowledged an informed consent statement attesting their participation in the study.

## Data availability

Data will be made available on request.

## Declaration of competing interest

The authors declare the following financial interests/personal relationships which may be considered as potential competing interests: Renata Ernlund Freitas de Macedo reports article publishing charges was provided by Coordenação de Aperfeiçoamento de Pessoal de Nível Superior - CAPES. If there are other authors, they declare that they have no known competing financial interests or personal relationships that could have appeared to influence the work reported in this paper.

## References

[1] Di Vita G., Blanc S., Mancuso T., Massaglia S., La Via G., D'Amico M. (2019). Harmful compounds and willingness to buy for reduced-additives salami. An outlook on Italian consumers. Int. J. Environ. Res. Publ. Health.

[2] Flores M., Olivares A. (2014). Handbook of Fermented Meat and Poultry.

[3] Boada L.D., Henríquez-Hernández L.A., Luzardo O.P. (2016). The impact of red and processed meat consumption on cancer and other health outcomes: epidemiological evidences. Food Chem. Toxicol..

[4] Martínez E., Pardo J.E., Álvarez-Ortí M., Rabadán A., Pardo-Giménez A., Alvarruiz A. (2023). Substitution of pork fat by emulsified seed oils in fresh deer sausage (‘Chorizo’) and its impact on the physical, nutritional, and sensory properties. Foods.

[5] Simunovic S. (2022). Reformulation of traditional fermented tea sausage utilizing novel (digital) methods of analysis. Foods.

[6] Based G., Cîrstea N., Nour V., Corbu A.R., Muntean C., Gabriela G. (2023). Reformulation of bologna sausage by total pork backfat. Foods.

[7] de Sousa A.M.B. (Sep. 2020). Storage of beef burgers containing fructooligosaccharides as fat replacer and potassium chloride as replacing sodium chloride. J. Food Sci. Technol..

[8] Zhang Y., Zhang Y., Zhou X., Wang S., Li P. (Mar. 2021). Salt replacement changed the bacterial community composition and physicochemical characteristics of sodium-reduced fermented sausages during fermentation and ripening. Foods.

[9] Vitola H.R.S., Dannenberg G.S., Marques J.L., Lopes G.V., Fiorentini A.M. (2018). Probiotic potential of Lactobacillus casei CSL3 isolated from bovine colostrum silage and its viability capacity immobilized in soybean. Process Biochem..

[10] Bis-Souza C.V., Barba F.J., Lorenzo J.M., Penna A.L.B., Barretto A.C.S. (2019). New strategies for the development of innovative fermented meat products: a review regarding the incorporation of probiotics and dietary fibers. Food Rev. Int..

[11] Mordor Intelligence LLP (Mar. 2019). Probiotics market - growth, trends, and forecast (2019 - 2024). http://www.mordorintelligence.com.

[12] Williams & Marshall Strategy Brazil Processed meat market grew at a 9.11% CAGR between 2015 and 2019. https://www.wm-strategy.com/news/brazil-processed-meat-market-2015-2019.

[13] Chen P.-J., Antonelli M. (2020). Conceptual models of food choice: influential factors related to foods, individual differences, and society. Foods.

[14] Hill C. (2014). Expert consensus document: the international scientific association for probiotics and prebiotics consensus statement on the scope and appropriate use of the term probiotic. Nat. Rev. Gastroenterol. Hepatol..

[15] Brasil, Agência Nacional de Vigilância Sanitária (ANVISA). Resolução da Diretoria Colegiada RDC n. 243 de 27 de julho de 2018 Dispõe sobre os requisitos sanitários dos suplementos alimentares,Brasília. Retrieved from ebeea49b-756b-4694-be94-5d2d57088018 (anvisa.gov.br), Accessed August 14, 2023.

[16] Herbert Stone H.A.T.-, Bleibaum Rebecca N. (2012). Sensory evaluation practices. https://books.google.com.br/books?hl=pt-BR&lr=&id=U2XRDwAAQBAJ&oi=fnd&pg=PP1&ots=q7nHyyGdG0&sig=Z97jfotNvRBAQBuZWcneafqia_w&redir_esc=y#v=onepage&q&f=false.

[17] Mark T.L., Swait J. (2004). Using stated preference and revealed preference modeling to evaluate prescribing decisions. Health Econ..

[18] Huffman W.E., McCluskey J.J. (2017). Using Stated preference techniques and experimental auction methods: a review of advantages and disadvantages for each method in examining consumer preferences for new technology. Int. Rev. Environ. Resour. Econ..

[19] Pires M.A., de Noronha R.L.F., Trindade M.A. (2019). Understanding consumer's perception and acceptance of bologna sausages with reduced sodium content and/or omega-3 addition through conjoint analysis and focus group. J. Sensory Stud..

[20] Polizer Rocha Y.J., de Noronha R.L.F., Trindade M.A. (2019). Relations between consumer's concern with own health and their perception about frankfurters with functional ingredients. Meat Sci..

[21] Viana M.M., dos Santos Silva V.L., Trindade M.A. (2014). Consumers' perception of beef burgers with different healthy attributes. Lwt.

[22] Schnettler B. (Jun. 2019). Are consumers willing to pay more for reformulated processed meat products in the context of the implementation of nutritional warnings? Case study with frankfurters in Chile. Meat Sci..

[23] Pontual I. (Apr. 2017). Assessing consumer expectations about pizza: a study on celiac and non-celiac individuals using the word association technique. Food Res. Int..

[24] Donoghue S. (2010). Projective techniques in consumer research. J. Fam. Ecol. Consum. Sci. /Tydskrif vir Gesinsekologie en Verbruikerswetenskappe.

[25] Viana M.M., Silva V.L.S., Deliza R., Trindade M.A. (2016). The use of an online completion test to reveal important attributes in consumer choice: an empirical study on frozen burgers. Food Qual. Prefer..

[26] Shan L.C., Henchion M., De Brún A., Murrin C., Wall P.G., Monahan F.J. (Nov. 2017). Factors that predict consumer acceptance of enriched processed meats. Meat Sci..

[27] Van Wezemael L., Verbeke W., De Barcellos M.D., Scholderer J., Perez-Cueto F. (2010). Consumer perceptions of beef healthiness: results from a qualitative study in four European countries. BMC Publ. Health.

[28] Di Vita G. (2022). The thin line between tradition and well-being: consumer responds to health and typicality attributes for dry-cured ham. J. Clean. Prod..

[29] Holck A., Axelsson L., Mcleod A., Rode T.M., Heir E. (2017). Health and safety considerations of fermented sausages. J. Food Qual..

[30] Shan L.C. (Sep. 2017). Consumer evaluations of processed meat products reformulated to be healthier – a conjoint analysis study. Meat Sci..

[31] Resano H., Pérez-Cueto F.J.A., Sanjuán A.I., de Barcellos M.D., Grunert K.G., Verbeke W. (2011). Consumer satisfaction with dry-cured ham in five European countries. Meat Sci..

[32] Delgado-Pando G., Ekonomou S.I., Stratakos A.C., Pintado T. (2021). Clean label alternatives in meat products. Foods.

[33] Clean Label Ingredient Market—Growth (2022).

[34] Banovic M., Reinders M.J., Claret A., Guerrero L., Krystallis A. (2019). A cross-cultural perspective on impact of health and nutrition claims, country-of-origin and eco-label on consumer choice of new aquaculture products. Food Res. Int..

[35] Verbeke W., Scholderer J., Lähteenmäki L. (Jun. 2009). Consumer appeal of nutrition and health claims in three existing product concepts. Appetite.

[36] Carfora V., Cavallo C., Catellani P., Del Giudice T., Cicia G. (Jun. 2021). Why do consumers intend to purchase natural food? Integrating theory of planned behavior, value-belief-norm theory, and trust. Nutrients.

[37] Eldesouky A., Pulido A.F., Mesias F.J. (2015). The role of packaging and presentation format in consumers' preferences for food: an application of projective techniques. J. Sensory Stud..

[38] Laaksonen O., Ma X., Pasanen E., Zhou P., Yang B., Linderborg K.M. (2020). Sensory characteristics contributing to pleasantness of oat product concepts by Finnish and Chinese consumers. Foods.

[39] Masson M., Delarue J., Bouillot S., Sieffermann J.M., Blumenthal D. (2016). Beyond sensory characteristics, how can we identify subjective dimensions? A comparison of six qualitative methods relative to a case study on coffee cups. Food Qual. Prefer..

[40] Roininen K., Lähteenmäki L., Tuorila H. (Aug. 1999). Quantification of consumer attitudes to health and hedonic characteristics of foods. Appetite.

[41] Deliza R., Rosenthal A., da Costa M.C. (2003). Tradução e validação para a língua portuguesa de questionário utilizado em estudos de consumidor. Ciência Tecnol. Aliment..

[42] Brazil (2021). “Lei n^o^ 14.158, de 2 de unho de 2021 Dispõe sobre o valor do salário-mínimo a vigorar a partir de 1^o^ de janeiro de 2021. Dispõe sobre o valor do salário-mínimo a vigorar a partir de 1^o^ de janeiro de 2021.,”.

[43] Bruschi V., Teuber R., Dolgopolova I. (2015). Acceptance and willingness to pay for health-enhancing bakery products - empirical evidence for young urban Russian consumers. Food Qual. Prefer..

[44] Timpanaro G., Bellia C., Foti V.T., Scuderi A. (2020). Consumer behaviour of purchasing biofortified food products. Sustain. Times.

[45] Bayton J.A. (1958). Motivation, cognition, learning—basic factors in consumer behavior. J. Market..

[46] Hao M., Wang W., Zhang J., Chen L. (2023). Flavour characteristics of fermented meat products in China: a review. Fermentation.

[47] Asioli D., Varela P., Hersleth M., Almli V.L., Olsen N.V., Næs T. (Mar. 2017). A discussion of recent methodologies for combining sensory and extrinsic product properties in consumer studies. Food Qual. Prefer..

[48] Castro I.A., Majmundar A., Williams C.B., Baquero B. (2018). Customer purchase intentions and choice in food retail environments: a scoping review. Int. J. Environ. Res. Publ. Health.

[49] Lorido L., Pizarro E., Estévez M., Ventanas S. (2019). Emotional responses to the consumption of dry-cured hams by Spanish consumers: a temporal approach. Meat Sci..

[50] Monsivais P., Aggarwal A., Drewnowski A. (2014). Time spent on home food preparation and indicators of healthy eating. Am. J. Prev. Med..

[51] Tarjuelo L., Rabadán A., Álvarez-Ortí M., Pardo-Giménez A., Pardo J.E. (2023). Analysis of nutritional characteristics and willingness to pay of consumers for dry-cured sausages (salchichón) made with textured seed oils. Foods.

[52] Di Vita G., Zanchini R., Spina D., Maesano G., La Via G., D'Amico M. (2022). Exploring purchasing determinants for a low fat content salami: are consumers willing to pay for an additional premium?. Front. Sustain. Food Syst..

[53] Kallas Z., Čandek-Potokar M., Tomažin U., Pugliese C., Aquilani C., Gil J.M. (2017). Measuring consumers' preferences for traditional and innovative pork products. Agric. Conspec. Sci..

[54] Schifferstein H.N.J., Oude Ophuis P.A.M. (May 1998). Health-related determinants of organic food consumption in The Netherlands. Food Qual. Prefer..

[55] Delgado-Pando G., Pintado T. (2022). New strategies for innovative and enhanced meat and meat products. Foods.

[56] Santiesteban-López N.A. (2022). Natural antimicrobials: a clean label strategy to improve the shelf life and safety of reformulated meat products. Foods.

[57] Solomando J.C. (2021). Front-of-pack nutrition labelling: testing effectiveness of different nutrition labelling formats front-of-pack in four European countries. Int. J. Food Sci. Technol..

[58] Feunekes G.I.J., Gortemaker I.A., Willems A.A., Lion R., van den Kommer M. (2008). Front-of-pack nutrition labelling: testing effectiveness of different nutrition labelling formats front-of-pack in four European countries. Appetite.

[59] Guerrero L. (2009). Consumer-driven definition of traditional food products and innovation in traditional foods. A qualitative cross-cultural study. Appetite.

[60] Sabbe S., Verbeke W., Deliza R., Matta V., Van Damme P. (Aug. 2009). Effect of a health claim and personal characteristics on consumer acceptance of fruit juices with different concentrations of açaí (Euterpe oleracea Mart.). Appetite.

[61] Shim S.M., Seo S.H., Lee Y., Moon G.I., Kim M.S., Park J.H. (Jul. 2011). Consumers' knowledge and safety perceptions of food additives: evaluation on the effectiveness of transmitting information on preservatives. Food Control.

[62] Shan L.C. (2016). Consumer views on ‘healthier’ processed meat. Br. Food J..

[63] Annunziata A., Vecchio R. (Apr. 2013). Consumer perception of functional foods: a conjoint analysis with probiotics. Food Qual. Prefer..

[64] Neves N.C.D.R., Roque-Specht Vã F., Gomes E.M.D.C. (2021). Functional foods: consumer perception in the federal district, Brazil. Mundo da Saude.

[65] Ares G., Gámbaro A. (Jul. 2007). Influence of gender, age and motives underlying food choice on perceived healthiness and willingness to try functional foods. Appetite.

[66] Hailu G., Boecker A., Henson S., Cranfield J. (Apr. 2009). Consumer valuation of functional foods and nutraceuticals in Canada. A conjoint study using probiotics. Appetite.

[67] Escaron A.L., Meinen A.M., Nitzke S.A., Martinez-Donate A.P. (2013). Supermarket and grocery store-based interventions to promote healthful food choices and eating practices: a systematic review. Prev. Chronic Dis..

[68] Raghunathan R., Naylor R.W., Hoyer W.D. (Oct. 2006). The Unhealthy = Tasty Intuition and Its Effects on Taste Inferences, Enjoyment, and Choice of Food Products.

