# Supplementary material for: Using online attitudinal and completion test to understand the consumerś perception of probiotic dry-fermented sausage
Source: Heliyon. 2024 Nov 28;10(23):e40738. doi: 10.1016/j.heliyon.2024.e40738 (PMC11650293; doi:10.1016/j.heliyon.2024.e40738)
Supplement: Multimedia component 1 [file mmc1.pdf]

ONLINE QUESTIONNAIRE REGARDING CONSUMERS´ PERCEPTION OF FUNCTIONAL DRY-FERMENTED SAUSAGE

INFORMED CONSENT FORM

* Indicates a mandatory question

Hello! You are being invited as a volunteer to participate in the study *"Relationship between health concerns and consumer perception of functional fermented sausage".

PARTICIPATION IN THE STUDY: Your participation in this study involves completing an online questionnaire hosted on the Google Docs platform, focusing on your sensory perceptions of dry-fermented sausage. You have the flexibility to respond at your convenience, following the provided instructions. The questionnaire comprises 6 sections and typically takes 5 to 10 minutes to complete.

RISK AND BENEFITS STATEMENT: In adherence to the Informed Consent Form, your participation solely aims to gather essential data for scientific purposes, strictly following the participant's consent. There are no direct health or integrity risks involved in your role. However, potential indirect risks may relate to discomfort or embarrassment while answering certain questions. To mitigate any potential embarrassment, your anonymity is ensured by not requiring personal identifiers (your name or email).

CONFIDENTIALITY AND PRIVACY ASSURANCE: We, as researchers, are committed to upholding your privacy throughout this study.

AUTONOMY AND WITHDRAWAL: You retain the autonomy to withdraw from the study at any time, whether by declining to participate initially or by retracting your consent without the need for explanation. Your data may be utilized to generate technical and scientific articles upon your authorization, maintaining your anonymity by not disclosing personal identifiers.

REFUND AND INDEMNITY POLICY: Your participation in this research is voluntary, and no compensation or expenses will be provided for your involvement. In the unlikely event of any harm resulting from the study, your indemnity will be ensured through reimbursement, compliant with legal requirements, via check issuance.

CONTACT INFORMATION: The researchers leading this project include Marilia Silva Malvezzi Karwowski, a PhD student in the Postgraduate Program in Animal Science at PUCPR; Renata Ernlund Freitas de Macedo, a full professor in the same program at PUCPR; and Evelin da Costa Boiko, a collaborating researcher with a post-graduate degree in Sanitary Surveillance and Quality Control Applied to Food Production at PUCPR. You can reach out

to them via the following phone numbers: +55 (41) 9 9619-7811, +55(41) 3271-2690, or +55 (41) 9 9557-2369, or through email at marilia_malvezzi@yahoo.com.br, renata.macedo@pucpr.br, evelinboiko@gmail.com.

ETHICS COMMITTEE INFORMATION: The Human Research Ethics Committee (CEP) comprises dedicated individuals striving to safeguard your rights as a research participant. Their responsibility includes evaluating the planning and execution of the research to ensure its appropriateness and adherence to ethical standards. In case you perceive that the research deviates from your expectations or if you believe you're encountering any form of harm, you have the option to contact the PUCPR Research Ethics Committee (CEP) by phone at +55 (41) 3271-2292, available Monday to Friday from 8:00 am to 5:30 pm, or via email at nep@pucpr.br.

DECLARATION: I affirm that I have thoroughly read and comprehended all the information provided in this Informed Consent Form. I have had the opportunity to discuss the contents of this form and all my inquiries have been sufficiently addressed, leaving me satisfied with the responses. I acknowledge that I will receive a signed and dated copy of this document for my records, while another signed and dated copy will be archived for the responsible researcher of this study. Having been properly briefed on the scope and implications of this research, and comprehending its nature and purpose, I voluntarily consent to participate. I understand and acknowledge that no monetary compensation is involved in my participation.

Do you agree to participate in this study?

Mark just one option.

- ◯ Yes, I agree to participate in the study, I am over 18 years old
- ◯ No, I do not agree and/or I' am under 18 years old *Skip for The section 6 (Acknowledgment)*

IDENTIFICATION

1 Which is your gender? *

Mark just one option.

- Female
- Male
- I prefer not to inform

1. In which state do you live? *

____

2. In which city do you live? *

____

3. With what frequency on average, do you consume fermented sausage, inside or outside your home? *

Mark just one option.

- Always (once a week or more)
- Frequently (2 or 3 times a month)
- Moderately (once a month)
- Sometimes (less than once a month)
- Rarely (only on special occasions)

CONSUMPTION PROFILE

4. Please, observe with attention the image below and fill in the sentence of balloon B (at the the field below the image) with the first 4 words or phrases that come from your mind. *

Figure 1: Image of a couple in the supermarket talking about buying fermented sausage.

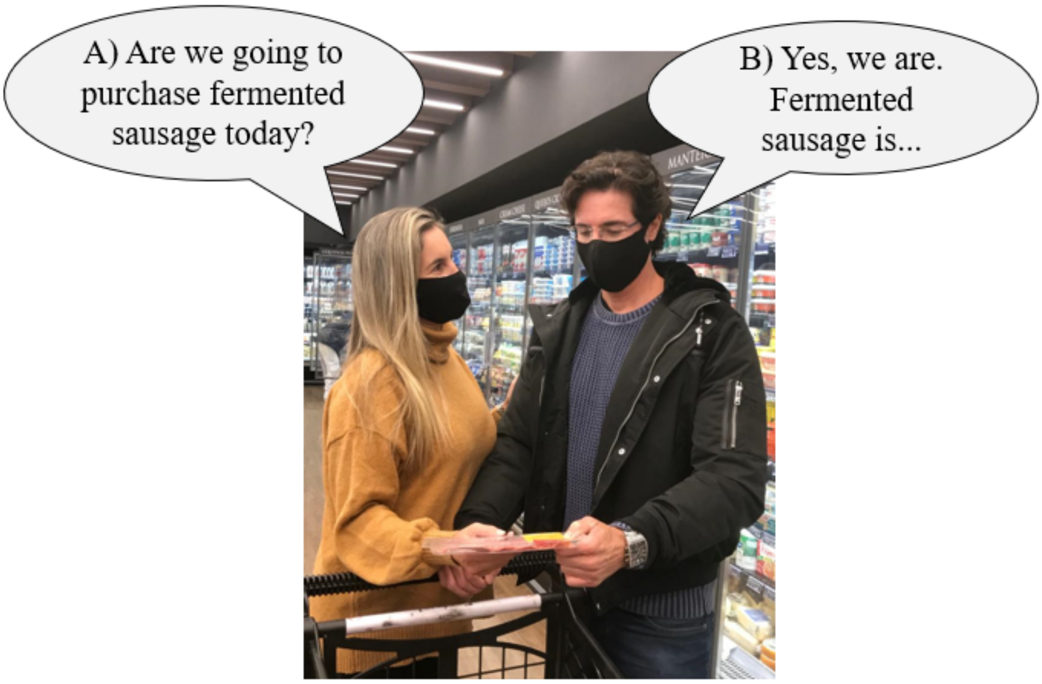

5. Please, observe with attention the image below and fill in the sentence of balloon B, at the the field below the image, with the first words or phrases that come from your mind. *

Figure 2: Image of a couple in the supermarket talking about buying fermented sausage.

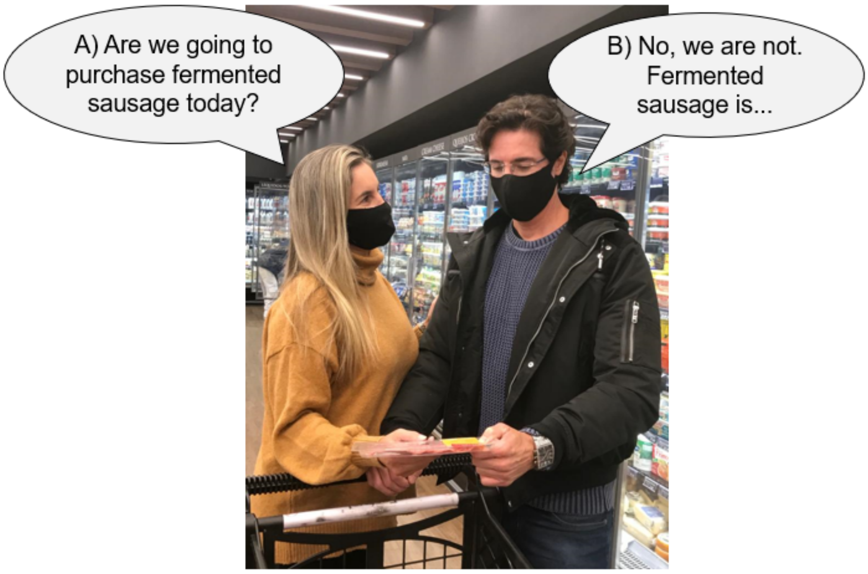

6. Please, observe with attention the image below and fill in the sentence of balloon B, at the the field below the image, with the first words or phrases that come from your mind. *

Figure 3: Image of a couple in the supermarket talking about buying functional fermented sausage.

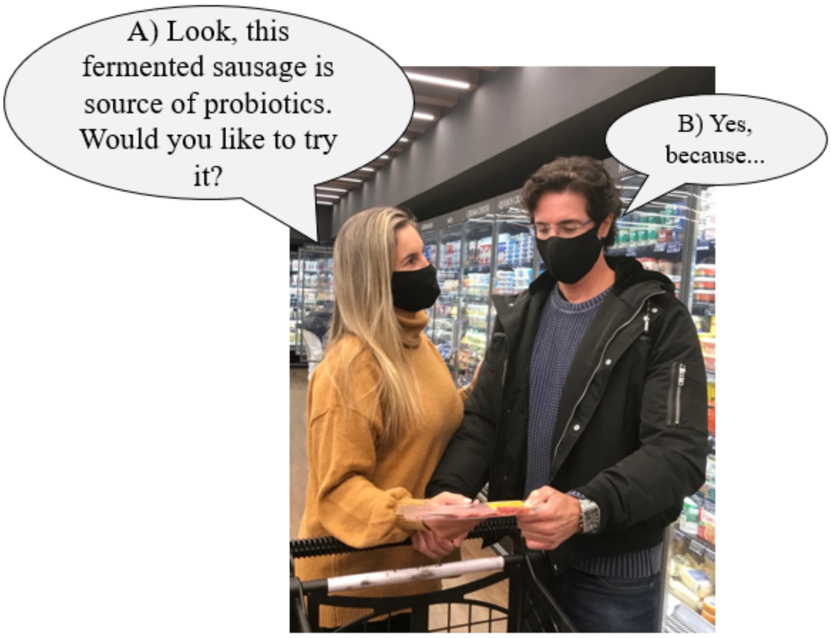

____

____

____

____

____

7. Please, observe with attention the image below and fill in the sentence of balloon B, at the the field below the image, with the first words or phrases that come from your mind. *

Figure 4: Image of a couple in the supermarket talking about buying functional fermented sausage.

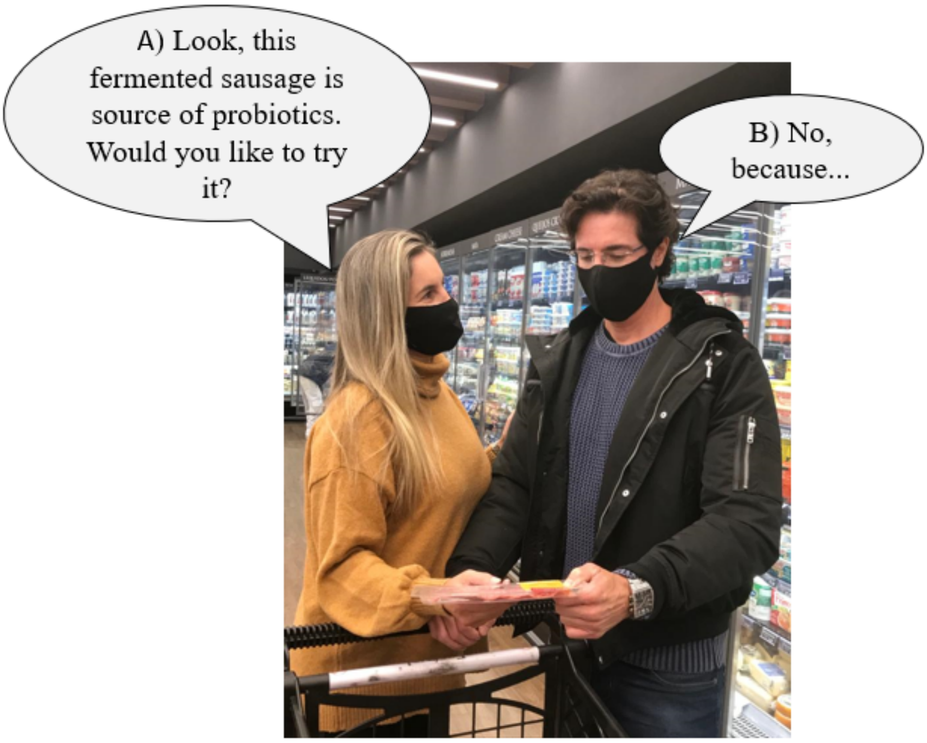

ATTITUDINAL PROFILE

8. Please, indicate the extent of your agreement with each of the following statements:

10.1 I have the impression that I sacrifice a lot for my health. *

Mark just one option.

	1	2	3	4	5	6	7	8	9	
I totally disagree	◯	◯	◯	◯	◯	◯	◯	◯	◯	I totally agree

10.2. I consider myself very health conscious. *

Mark just one option.

	1	2	3	4	5	6	7	8	9	
I totally disagree	◯	◯	◯	◯	◯	◯	◯	◯	◯	I totally agree

10.3. I am prepared to sacrifice a lot, to eat as healthy as possible.*

Mark just one option.

	1	2	3	4	5	6	7	8	9	
I totally disagree	◯	◯	◯	◯	◯	◯	◯	◯	◯	I totally agree

10.4. I think that I take health into account a lot in my life.*

Mark just one option.

	1	2	3	4	5	6	7	8	9	
I totally disagree	◯	◯	◯	◯	◯	◯	◯	◯	◯	I totally agree

10.5. I think it is important to know how to eat healthily.*

Mark just one option.

	1	2	3	4	5	6	7	8	9	
I totally disagree	◯	◯	◯	◯	◯	◯	◯	◯	◯	I totally agree

10.6. My health is so valuable to me that I am prepared to sacrifice many things for it.*

Mark just one option.

	1	2	3	4	5	6	7	8	9	
I totally disagree	◯	◯	◯	◯	◯	◯	◯	◯	◯	I totally agree

10.7. I have the impression that other people pay more attention to their health than I do to mine *

Mark just one option.

	1	2	3	4	5	6	7	8	9	
I totally disagree	◯	◯	◯	◯	◯	◯	◯	◯	◯	I totally agree

10.8. I do not continually ask myself whether something is good for me.*

Mark just one option.

	1	2	3	4	5	6	7	8	9	
I totally disagree	◯	◯	◯	◯	◯	◯	◯	◯	◯	I totally agree

10.9. I don't often think about whether everything I do is healthy.*

Mark just one option.

	1	2	3	4	5	6	7	8	9	
I totally disagree	◯	◯	◯	◯	◯	◯	◯	◯	◯	I totally agree

10.10. I don't want to ask myself all the time whether the things I eat are good for me *

Mark just one option.

	1	2	3	4	5	6	7	8	9	
I totally disagree	◯	◯	◯	◯	◯	◯	◯	◯	◯	I totally agree

10.11. I often dwell on my health.*

Mark just one option.

	1	2	3	4	5	6	7	8	9	
I totally disagree	◯	◯	◯	◯	◯	◯	◯	◯	◯	I totally agree

SOCIOECONOMIC PROFILE

9. How old are you? *

Mark just one option.

- ◯ 18 - 25 years
- ◯ 26 - 35 years
- ◯ 36 - 45 years
- ◯ 46 - 56 years
- ◯ 57 - 70 years
- ◯ over 70 years

10. What is your education level? *

To mark just one oval.

- ( ) Secondary incomplete
- ( ) Secondary
- ( ) Undergraduate incomplete
- ( ) Undergraduate
- ( ) Graduate

11. Adding your income to the income of people who live with you, *approximately how much is your monthly family income?

Mark just one option.

- ( ) ≤ US$ 440.00
- ( ) ≥ US$ 440.01 ≤ US$ 896.00
- ( ) ≥ US$ 896.01≤ US$ 2,200.00
- ( ) ≥ US$ 2,200.01 ≤ US$ 4,400.00
- ( ) > US$ 4,400.01

ACKNOWLEDGMENT

Thank you for your participation.
Yours sincerely,

Evelin from the Coast Boiko
Marilia Malvezzi Karwowski
PUCPR

Google Forms
